# Lifetime risk of herpes zoster in the population of Beijing, China

**DOI:** 10.1016/j.puhip.2023.100356

**Published:** 2023-01-10

**Authors:** Christa Lee, Nikolaos Giannelos, Desmond Curran, Hengjin Dong, Haiwen Tang, Ning Jiang, Chiyu Ye, Yanfei Yuan, Cheryl Ng

**Affiliations:** a GSK, Singapore, Singapore; b GSK, Wavre, Belgium; c Center for Health Policy Studies, School of Public Health, Zhejiang University School of Medicine, Hangzhou, China; d GSK, Beijing, China

**Keywords:** Aging population, China, Herpes zoster, Lifetime risk, Postherpetic neuralgia, Vaccination

## Abstract

**Objectives:**

We aimed to estimate the current and future lifetime risks (LTR) of herpes zoster (HZ) and postherpetic neuralgia (PHN), as well as their respective number of annual incident cases in Beijing, China, if individuals were not vaccinated against HZ.

**Study design:**

Mathematical model built in Microsoft Excel, *de novo*.

**Methods:**

A hypothetical cohort of 1,000 people was simulated from age 0–100 or until death to generate LTRs of HZ/PHN in Beijing, China. LTR was defined as the risk of developing HZ/PHN at least once in the person’s lifetime. The current number of annual incident HZ/PHN cases were also calculated by multiplying up-to-date population data and the annual age-specific incidence of HZ/PHN. For both LTR and annual incident cases, current estimates were projected into the year 2035 to investigate the impact of an aging population. Scenario and deterministic sensitivity analyses (DSA) were conducted to validate the model outcomes.

**Results:**

In Beijing, the current and future LTRs of HZ (PHN) were 32.4% (2.8%) and 34.8% (3.3%), respectively. The current and future annual incident cases of HZ (PHN) of individuals aged ≥50 years were 68,394 (7,801) cases among 7.04 million individuals and 88,676 (9,649) cases among 9.08 million individuals, respectively. The scenario analyses demonstrated that modelled results were likely to underestimate the LTR of HZ. Results were robust under the DSA.

**Conclusions:**

Given an aging population, HZ poses a significant, growing burden on individuals, the society, and healthcare system of China, highlighting the need for preventative measures such as vaccination.

## Graphical abstract

**Figure:**
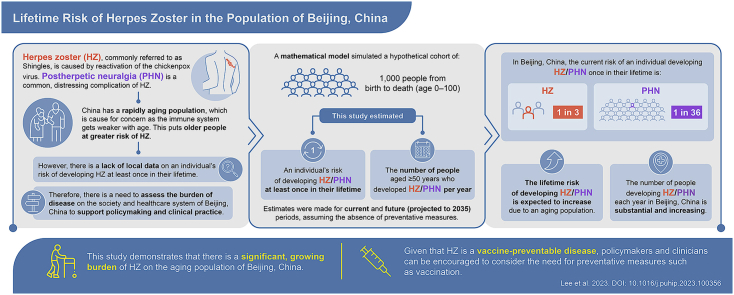

## 1 Introduction

Herpes zoster (HZ), commonly referred to as shingles, is caused by reactivation of a latent varicella zoster virus (VZV) infection when VZV-specific cell-mediated immunity falls below a critical threshold [1]. Aging is associated with a degree of immunosuppression, and an age-related decline in immunity (ARDI) translates to an increasing risk of HZ with age [2,3].

HZ is characterised by a painful, dermatomal vesicular rash that often resolves within 2–4 weeks [2]. While most cases are self-limiting, a proportion of HZ cases may develop complications. The most common complication of HZ is postherpetic neuralgia (PHN), a persistent, debilitating pain that can last from several months to years [2,4,5]. PHN affects 5–30% of individuals with HZ, and its risk also increases with age [6]. Other HZ-associated complications include ocular and neurological involvement, all of which may result in long-term physical impairment [4,5]. Consequently, HZ and its associated complications can greatly reduce an individual’s overall quality of life, as well as having a spill-over effect on the affected individual’s carers [5,7].

Recent systematic literature reviews across multiple regions (including North America, Europe, and Asia-Pacific) have reported comparable HZ incidence to that of mainland China [6,8,9]. Previous studies conducted in the North American region, United Kingdom, and Taiwan have estimated the risk of developing HZ at least once in an individual’s lifetime to be approximately 30% (1 in 3 individuals) [2,10,11]. Though previously thought to be a once-in-a-lifetime experience for most, HZ has been observed to recur even among immunocompetent individuals; a study in the United States estimated the 8-year population-based recurrence rate of HZ as 6.2%, with recurrences being as common as the initial risk of developing HZ when matched by sex, age, and immune status (immunocompromised or immunocompetent) [12].

However, there is still a paucity of data on the lifetime risk of developing HZ in mainland China. Moreover, China’s rapidly growing population of older adults presents a key challenge to the country [9,13], given that ARDI is known to increase the risk of HZ [2,3]. This obstacle is compounded by the limitations of current HZ therapies, which do not necessarily guarantee a reduction in HZ and PHN-associated morbidity [14,15]. In light of these limitations, data on the lifetime risk of HZ can help to support policymakers and clinicians in evaluating the value of preventive measures against HZ in China, which may be of particular benefit to older adults [14].

This modelling exercise therefore aimed to address a gap in the literature by estimating the current and future lifetime risks (projected to the year 2035 to account for an aging demographic) of HZ and PHN in Beijing, China, in the absence of preventative measures. The current and future number of annual incident cases were additionally calculated, to contextualise the lifetime risk estimates.

## 2 Methods

### 2.1 Model overview

A mathematical model was built in Microsoft Excel, *de novo,* to simulate a hypothetical cohort of 1,000 individuals, simulated from age 0–100 or death, whichever was earlier. In brief, each cycle in the model corresponded with 1 year of life, and it was assumed that no subjects remained alive after 100 cycles (100 years). Cohort demographic information are described in detail in the ‘Demographics’ section.

**Fig. 1:**
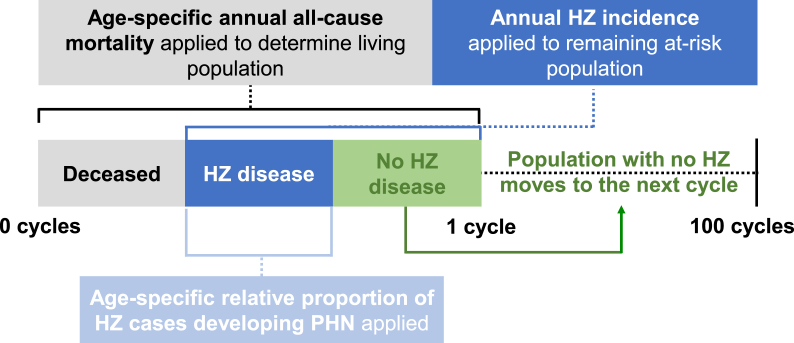
Model design. A cohort of 1,000 people was followed-up from age 0 to end-of-life (after 100 cycles, it was assumed that no subjects remained alive). 1 cycle corresponded with 1 year of life. Individuals previously infected with HZ were excluded from the next cycle. Age-specific HZ and PHN cases were summed up respectively and divided by the starting cohort of 1,000 to generate the respective overall lifetime risks. HZ: herpes zoster; PHN: postherpetic neuralgia.

First, age-specific annual all-cause mortality was applied to determine the living population. Subsequently, age-specific annual HZ incidence was applied to the remaining at-risk population to determine the proportion of individuals with a first episode of HZ. The living population who had not been infected with HZ in the previous cycle then moved to the next cycle. To calculate the number of age-specific PHN cases, the age-specific relative proportion of HZ cases developing PHN was applied to those that had developed HZ. Age-specific HZ and PHN cases were summed up respectively and divided by the starting cohort (1,000 individuals) to generate the respective overall lifetime risks. The full modelling design is presented in Fig. 1.

HZ disease burden was estimated and projected under the assumption that the local population had no access to any HZ vaccine, although the National Medical Products Administration approved the adjuvanted recombinant zoster vaccine (RZV, *Shingrix,* GSK) for China for preventative use in adults aged ≥50 years in May 2019. The model also assumed that childhood VZV vaccination was unlikely to have a significant impact on the analysis, as current data on its influence on an individuals’ lifetime risk of HZ are limited and inconclusive (Supplementary Appendix 1) [[16], [17], [18], [19], [20], [21], [22]].

### 2.2 Model inputs

Structured literature reviews and manual searches were undertaken in English and Chinese to identify relevant model inputs and support assumptions. Beijing was the primary focus of the modelling exercise, given its significance as China’s capital and for data availability. Hence, the model utilised Beijing-specific data where possible. In the absence of such data, estimates from mainland China or from other Chinese cities with similar demographic profiles to Beijing were used.

#### 2.2.1 Demographics

Current population estimates for the target population (aged ≥50 years) were derived from the most up-to-date estimates in the 2020 Beijing Statistical Yearbook (Table S1) [23]. The future population estimate for year 2035 was then obtained by summing per year from 2014–2019 (excluding 2010–2013 due to excessive fluctuation) and applying an exponential smoothing algorithm to provide a more conservative estimate compared with a linear projection. The year 2035 was chosen for future estimates to ensure relevancy of forecasted data.

The future population estimate for individuals aged ≥50 years in Beijing was validated by comparing the differences in population size between 2015 and 2035 for this modelling exercise and mainland China. In comparison with an overall population growth of 1.54 times as reported by the United Nations World Population Prospects (UNWPP) for mainland China, the model reported a lower population multiplier of 1.38, thereby demonstrating a likely conservative approach to projections of a rapidly aging population in Beijing.

#### 2.2.2 All-cause mortality

Given the absence of Beijing-specific all-cause mortality data, current and future mortality estimates were obtained from the 2015–2020 and 2035–2040 UNWPP 2019 life tables, respectively, for mainland China (Table S2) [24]. Medium-variant projection was used for future mortality estimates, which accounted for median fertility, normal mortality, and normal international migration rates [25]. This was expected to result in a conservative analysis outcome, given that the average life expectancy of Beijing is generally higher than that for mainland China overall [23,24,26].

#### 2.2.3 Incidence of HZ and PHN

Jiang et al., 2019 was used as the primary data source for HZ and PHN incidence in this model, as it provides baseline data on the disease burden of HZ in a similarly urbanised city in China [27]. The average incidence of HZ and the proportion of HZ cases with PHN were stratified by age group (Table S3 and Table S4). Due to the challenges of predicting projected incidence and an absence of these predictions in existing literature, a conservative approach was used to avoid over-estimation of risk. A last observation carried forward was therefore assumed for both age-specific HZ and PHN incidence, implying that incidence remained constant throughout the projection period as the cohort aged over time. Consequently, any change in HZ disease burden over time would be attributable the effect of demographic changes [28].

### 2.3 Outcomes

#### 2.3.1 Current and future lifetime risk estimates

Lifetime risk was defined as the risk of developing HZ or PHN at least once in a person’s lifetime. For base case results, current and future (projected to 2035) lifetime risk estimates of HZ and PHN were reported. These results were subsequently validated using probabilistic lifetime risk calculations.

#### 2.3.2 Annual number of incident cases

The annual number of incident HZ and PHN cases among individuals aged ≥50 years were estimated by multiplying the most up-to-date population data or its projection to 2035 with the corresponding value of the annual age-specific incidence of HZ and PHN. Data on the annual number of incident cases were only reported for individuals aged ≥50 years given the disease burden in older adults, as shown by the 2-fold increase in average HZ incidence between individuals aged 40–49 years to those aged 50–59 years (Table S3). Stratification of data beginning at 50 years was therefore considered to be the most relevant age cut-off.

### 2.4 Statistical considerations

#### 2.4.1 Probabilistic lifetime risk calculations

Base case results were validated using probabilistic lifetime risk calculations, which assessed the likelihood of an individual developing HZ or PHN up till life expectancy for current and future periods, independent of all-cause mortality data. This approach was also used to calibrate the mortality multipliers used in the scenario and sensitivity analyses of base case results.

$$risk=1-\prod_{n=0}^{LE-1}\left(1-\alpha_{n}\right)$$

Where *n* denotes age, *LE* denotes the average life expectancy, and *α* denotes the age-specific annual probability of an individual developing HZ.

#### 2.4.2 Scenario analyses

##### 2.4.2.1 Lower mortality

Mortality multipliers of 0.6 and 0.5 were applied to provide a more realistic portrayal of current and future lifetime risks of HZ in the base case, respectively. This scenario analysis was conducted to account for a higher life expectancy in Beijing compared with mainland China [23,24,26], as the use of UNWPP-reported mortalities for mainland China are likely to result in underestimated lifetime risks for Beijing.

##### 2.4.2.2 Remaining lifetime risk at stratified age cut-offs

Current remaining lifetime risk estimates of HZ were calculated in a second scenario analysis. Remaining lifetime risk was defined as the risk of developing HZ at least once in an individual’s lifetime from the base case scenario, but starting at age cut-offs of interest; specifically, cut-offs were set at 50, 60, 65 and 70 years to investigate the disease burden among older adults. Future remaining lifetime risk estimates at these age cut-offs were not reported, as a similar trend in results was observed.

##### 2.4.2.3 Progressive all-cause mortalities

Two UNWPP life tables (2015–2020 for current and 2035–2040 for future, respectively) were used for base case lifetime risk estimates to accommodate a straightforward modelling approach. However, this would not capture declining mortality estimates when projected into the future, which may therefore underestimate lifetime risk. Hence, a third scenario analysis was conducted to provide a more realistic depiction of current remaining lifetime risk estimates among older adults, given the relatively substantial disease burden in this age group. Progressively decreasing all-cause mortalities were applied to each age group (50, 60, 65, and 70 years) from the remaining lifetime risk scenario analysis; all-cause mortality data were updated in 5-year increments, based on multiple UNWPP life tables spanning 2015–2020 and 2045–2050.

#### 2.4.3 Sensitivity analysis

A deterministic sensitivity analysis (DSA) was conducted to assess the robustness of the overall current and future lifetime risks associated with HZ. Briefly, the model’s key input variables were individually varied within ±20% of the base case (full list of parameters detailed in Table S5). The lower-bound values for the mortality multiplier parameters were calibrated using previously described probabilistic lifetime risk calculations. On the other hand, upper-bound values were set at 1 owing to the higher life expectancy in Beijing compared with mainland China [23,24,26].

## 3 Results

### 3.1 Base case results

**Fig. 2:**
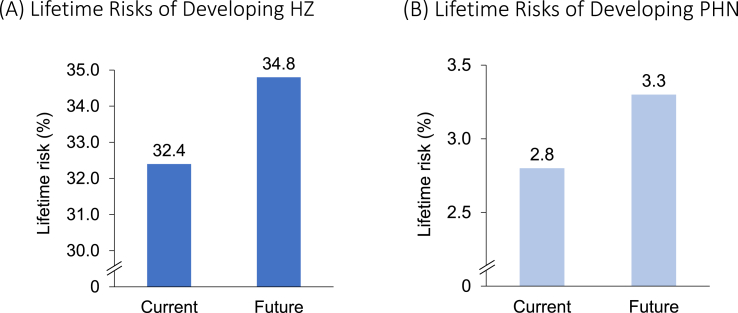
Base case results: Lifetime risks of developing HZ and PHN HZ: herpes zoster; PHN: postherpetic neuralgia.

In Beijing, China, the current lifetime risk of HZ (PHN) was 32.4% (2.8%) (Fig. 2). The future lifetime risk of HZ (PHN) was projected to be 34.8% (3.3%), a relative increase of 7.5% (16.8%) from current risk estimates. Probabilistic lifetime risk estimates for current (32.5% [2.8%]) and future (34.8% [3.2%]) were similar, thereby validating the model’s results. Annual incident cases of HZ (PHN) in Beijing, China among 7.04 million individuals aged ≥50 years were reported to be 68,394 (7,801) in 2019, which was projected to increase to 9.08 million individuals and 88,676 (9,649) cases among the same age group in 2035 (an increase of 29.7% [23.4%]).

### 3.2 Scenario analyses

#### 3.2.1 Lower mortality

**Fig. 3:**
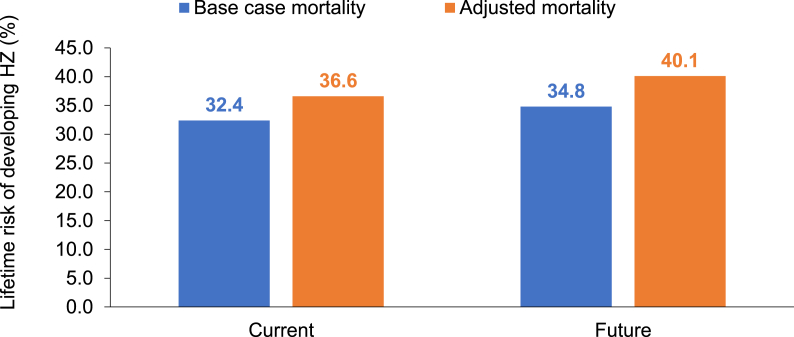
Applying lower mortality to base case scenario lifetime risk estimates of HZ HZ: herpes zoster.

Current lifetime risk of HZ was higher in the adjusted mortality scenario analysis (36.6%) compared with base case mortality (32.4%) (Fig. 3). A similar trend was observed for future lifetime risk estimates of HZ between adjusted mortality scenario analysis (40.1%) and base case mortality (34.8%).

#### 3.2.2 Remaining lifetime risk at stratified age cut-offs using base-case and progressive all-cause mortalities

The current remaining lifetime risk of HZ at age 50, 60, 65, and 70 years was 24.7%, 19.4%, 16.2%, and 13.4%, respectively. Application of progressive all-cause mortalities to age-stratified current remaining lifetime risk data increased the remaining lifetime risk of HZ at age 50, 60, 65, and 70 years to 27.0%, 20.9%, 17.2%, and 14.0%, respectively.

### 3.3 Sensitivity analysis

**Fig. 4:**
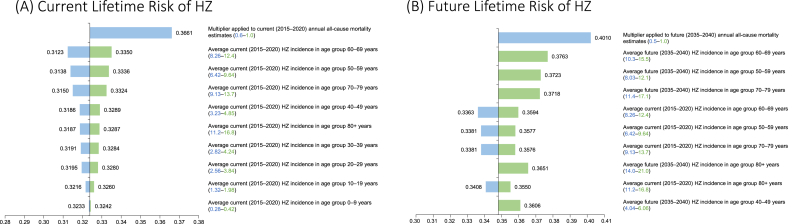
Deterministic sensitivity analysis The top 10 most influential parameters in the deterministic sensitivity analysis are presented. Lower bound estimates are in blue and upper bound estimates are in green, with lower and upper ranges for inputs in brackets. The centre line represents the base case values of 32.4% and 34.8% for (A) current lifetime risk of HZ and (B) future lifetime risk of HZ, respectively. HZ: herpes zoster.

Overall, results were robust under the DSA. Current and future lifetime risks of HZ varied between 31.2–36.6% and 33.6–40.1%, respectively. All-cause mortality was the parameter with the highest impact on HZ lifetime risk estimation, with longer life expectancy driving a higher lifetime risk of HZ. HZ incidence was the second-most influential parameter in the DSA. The top 10 most influential parameters for current and future lifetime risks of HZ are illustrated in Fig. 4.

## 4 Discussion

This modelling exercise estimated that 1 in 3 (32.4%) and 1 in 36 (2.8%) individuals from Beijing, China will develop HZ and PHN in their lifetime, respectively. The remaining lifetime risk of HZ remained high among individuals aged 50 years and above, suggesting a high disease burden among this age group. Furthermore, lifetime risk estimates of HZ and PHN were also both observed to increase when projected into the future, which may be attributable to longer life expectancy, resulting in a larger composition of greater-risk older age groups. The significant and increasing number of annual incident cases of HZ and PHN in Beijing similarly support this notion of an at-risk aging population. The relationship between longer life expectancy and higher lifetime risks of HZ is further reinforced by results from the DSA, which showed that lifetime risk estimates were most impacted by lower all-cause mortality.

Results from this modelling exercise are in line with previous research in other countries, which have similarly estimated a 1 in 3 lifetime risk of HZ [2,10,11], and also aligns with lifetime risk estimates delineated by country-specific HZ guidelines [[29], [30], [31], [32]]. Yet, the true lifetime risk of HZ in Beijing may still be underestimated, as the authors took a conservative approach. In reality, a higher lifetime risk of HZ could correspond with a disease burden that is greater than expected, given Beijing’s rapidly aging population. These findings collectively suggest that the already significant burden of HZ on older individuals, the society, and the healthcare system of Beijing, China is growing and will continue to grow.

These findings contribute towards overall long-term preventative strategies against HZ to avoid the growing burden of this disease. For example, local clinicians can be encouraged to consider vaccination as one such strategy, where they can hold proactive discussions with patients on their risk of HZ and recommend vaccination in line with local consensus statements or guidelines. At a policymaker level, expanding access to safe and efficacious vaccines that can confer long-term protection against HZ may also be considered to support and complement these local practices.

To the authors’ knowledge, this modelling exercise represents the first of its kind in examining the lifetime risk of HZ in Beijing, China. This is also the only publication, globally, to have estimated lifetime risk of HZ in the last 10 years. Furthermore, the modelling approach provided a robust methodology compared with existing publications on lifetime risk of HZ, which mainly applied formulae to derive an overall estimate [2,10,11]. Moreover, the *de novo* inception of the model enabled predictions for both current and future lifetime risk estimates of HZ, the latter of which is key to evaluating the influence of an aging population. These findings therefore serve as a key update to the epidemiology of HZ while simultaneously bridging a localised gap in current literature, and may also guide future investigations. Despite a Beijing-specific population, findings may be applicable to other urban cities in China with comparable HZ incidence and all-cause mortality.

On the other hand, a limitation of the modelling exercise related to the lack of Beijing- or China-specific data, as well as exhaustive, in-depth data to support model inputs and guide projections. For instance, baseline HZ incidence data from Yichang may not be fully representative of Beijing, given the differences in economic level and demographic structure between the two cities. To combat this, HZ incidence parameters were varied in the deterministic sensitivity analyses. Additionally, a probabilistic lifetime risk estimation was undertaken to mitigate limitations associated with all-cause mortality data and validate findings. Key parameters were also varied in both scenario and sensitivity analyses to capture uncertainty.

Finally, it may also be questioned why HZ lifetime risk estimates from this model were similar to, and not higher than, international findings from older publications [2,10,11], despite a global trend of increasing HZ incidence among all age strata [28,[33], [34], [35], [36], [37], [38]]. This may be attributable to differences in mortality between different countries, as well as under-reporting of HZ incidence in China. The model additionally assumed a constant incidence as a conservative approach, to avoid over-estimations of risk.

In conclusion, data from this modelling exercise establish the significant, growing burden of HZ in Beijing, China. These results highlight the need for proactive, preventative measures, such as vaccination, to alleviate this burden on the rapidly aging population and healthcare system of the country.

## Statement of ethical approval

This article is based on mathematical modelling with inputs informed primarily by previously conducted studies and does not contain any studies with human participants or animals performed by any of the authors.

## Funding

This study was sponsored by GlaxoSmithKline Biologicals SA (Study identifier eTrack VEO-000051). Support for third-party writing assistance for this article, provided by Paige Foo Jia-Qi, Costello Medical, Singapore, was funded by GSK in accordance with Good Publication Practice (GPP3) guidelines (http://www.ismpp.org/gpp3).

## Data availability

All data generated or analysed during this study are included in this published article/as supplementary information files.

## Trademark statement

Shingrix is a trademark of the GSK group of companies.

## Prior presentation

This article includes data that have been presented as a poster at the 17^th^ Annual Meeting of China Dermatologist Association and National Congress of Cosmetic Dermatology (CDA), 9–12 December 2021, China.

## Declarations of competing interest

NG, DC, HT, CY, and CN are employed by the GSK group of companies. CL, NJ, and YY were employees of the GSK group of companies at the time the study was conducted. HD reports nothing to disclose. The final article has been approved by all authors.

## References

[1] Chlibek R., Pauksens K., Rombo L., van Rijckevorsel G., Richardus J.H., Plassmann G. (2016). Long-term immunogenicity and safety of an investigational herpes zoster subunit vaccine in older adults. Vaccine.

[2] Harpaz R., Ortega-Sanchez I.R., Seward J.F. (2008). Prevention of herpes zoster: recommendations of the advisory committee on immunization practices (ACIP). MMWR Recomm. Rep. (Morb. Mortal. Wkly. Rep.).

[3] Yawn B.P., Gilden D. (2013). The global epidemiology of herpes zoster. Neurology.

[4] Dworkin R.H., Johnson R.W., Breuer J., Gnann J.W., Levin M.J., Backonja M. (2007). Recommendations for the management of herpes zoster. Clin. Infect. Dis..

[5] Johnson R.W., Bouhassira D., Kassianos G., Leplège A., Schmader K.E., Weinke T. (2010). The impact of herpes zoster and post-herpetic neuralgia on quality-of-life. BMC Med..

[6] Kawai K., Gebremeskel B.G., Acosta C.J. (2014). Systematic review of incidence and complications of herpes zoster: towards a global perspective. BMJ Open.

[7] Yu S.Y., Fan B.F., Yang F., DiBonaventura M., Chen Y.X., Li R.Y. (2019). Patient and economic burdens of postherpetic neuralgia in China. Clinicoecon. Outcomes Res..

[8] van Oorschot D., Vroling H., Bunge E., Diaz-Decaro J., Curran D., Yawn B. (2021). A systematic literature review of herpes zoster incidence worldwide. Hum. Vaccines Immunother..

[9] Yin D., Van Oorschot D., Jiang N., Marijam A., Saha D., Wu Z. (2021). A systematic literature review to assess the burden of herpes zoster disease in China. Expert Rev. Anti Infect. Ther..

[10] Brisson M., Edmunds W.J., Law B., Gay N.J., Walld R., Brownell M. (2001). Epidemiology of varicella zoster virus infection in Canada and the United Kingdom. Epidemiol. Infect..

[11] Lin Y.H., Huang L.M., Chang I.S., Tsai F.Y., Lu C.Y., Shao P.L. (2010). Disease burden and epidemiology of herpes zoster in pre-vaccine Taiwan. Vaccine.

[12] Yawn B.P., Wollan P.C., Kurland M.J., St Sauver J.L., Saddier P. (2011). Herpes zoster recurrences more frequent than previously reported. Mayo Clin. Proc..

[13] Fang E.F., Scheibye-Knudsen M., Jahn H.J., Li J., Ling L., Guo H. (2015). A research agenda for aging in China in the 21st century. Ageing Res. Rev..

[14] Cunningham A.L., Breuer J., Dwyer D.E., Gronow D.W., Helme R.D., Litt J.C. (2008). The prevention and management of herpes zoster. Med. J. Aust..

[15] Chen L.-K., Arai H., Chen L.-Y., Chou M.-Y., Djauzi S., Dong B. (2017). Looking back to move forward: a twenty-year audit of herpes zoster in Asia-Pacific. BMC Infect. Dis..

[16] Centers for Disease Control and Prevention: National Center for Immunization and Respiratory Diseases. Advisory Committee on Immunization Practices The Impact of the U.S. Varicella Vaccination Program on the Incidence of Herpes Zoster. https://stacks.cdc.gov/view/cdc/58913/cdc_58913_DS1.pdf?download-document-submit=Download.

[17] (2014). Varicella and herpes zoster vaccines: WHO position paper, June 2014 – Recommendations. Vaccine.

[18] Carryn S., Cheuvart B., Povey M., Dagnew A.F., Harpaz R., van der Most R. (2021). No consistent evidence of decreased exposure to varicella-zoster virus among older adults in countries with universal varicella vaccination. J. Infect. Dis..

[19] Gershon A.A., Gershon M.D. (2022). Widespread use of varicella vaccine does not reduce immunity to zoster of others. J. Infect. Dis..

[20] Harpaz R., Leung J.W. (2018). The epidemiology of herpes zoster in the United States during the era of varicella and herpes zoster vaccines: changing patterns among children. Clin. Infect. Dis..

[21] Wolfson L.J., Daniels V.J., Altland A., Black W., Huang W., Ou W. (2020). The Impact of varicella vaccination on the incidence of varicella and herpes zoster in the United States: updated evidence from observational databases, 1991-2016. Clin. Infect. Dis..

[22] Harpaz R., Leung J.W. (2019). The epidemiology of herpes zoster in the United States during the era of varicella and herpes zoster vaccines: changing patterns among older adults. Clin. Infect. Dis..

[23] China Statistics Press (2020). http://nj.tjj.beijing.gov.cn/nj/main/2020-tjnj/zk/indexeh.htm.

[24] United Nations: World Population Prospects (2019). Probabilistic Population Projections.

[25] United Nations Department of Economic and Social Affairs: World Population Prospects (2019). Definition of Project Variants.

[26] Xinhua (2018). Beijing’s Life Expectancy Rises to 82.15 Years.

[27] Jiang W., Li G., Xu Y., Pei S., Tong H., Wang W. (2019). Epidemiological characteristics of herpes zoster in urban areas of Yichang city during 2016-2017 based on the Yichang big data platform for health management. Chin. J. Vaccines Immunization.

[28] Varghese L., Standaert B., Olivieri A., Curran D. (2017). The temporal impact of aging on the burden of herpes zoster. BMC Geriatr..

[29] Centers for Disease Control and Prevention (2021). https://www.cdc.gov/vaccines/pubs/pinkbook/herpes-zoster.html.

[30] National Institute for Health and Care Excellence: Clinical Knowledge Summaries Shingles. https://cks.nice.org.uk/topics/shingles/background-information/prevalence/.

[31] Australian Government: Department of Health Shingles (Herpes Zoster). https://www.health.gov.au/health-topics/shingles-herpes-zoster.

[32] Ministry of Health of New Zealand Zoster (herpes zoster/shingles). https://www.health.govt.nz/our-work/immunisation-handbook-2020/23-zoster-herpes-zoster-shingles.

[33] Chao D.Y., Chien Y.Z., Yeh Y.P., Hsu P.S., Lian I.B. (2012). The incidence of varicella and herpes zoster in Taiwan during a period of increasing varicella vaccine coverage, 2000-2008. Epidemiol. Infect..

[34] Leung J., Harpaz R., Molinari N.A., Jumaan A., Zhou F. (2011). Herpes zoster incidence among insured persons in the United States, 1993-2006: evaluation of impact of varicella vaccination. Clin. Infect. Dis..

[35] MacIntyre R., Stein A., Harrison C., Britt H., Mahimbo A., Cunningham A. (2015). Increasing trends of herpes zoster in Australia. PLoS One.

[36] Marra F., Chong M., Najafzadeh M. (2016). Increasing incidence associated with herpes zoster infection in British Columbia, Canada. BMC Infect. Dis..

[37] Toyama N., Shiraki K. (2018). Universal varicella vaccination increased the incidence of herpes zoster in the child-rearing generation as its short-term effect. J. Dermatol. Sci..

[38] Curran D., Callegaro A., Fahrbach K., Neupane B., Vroling H., van Oorschot D. (2022). Meta-regression of herpes zoster incidence worldwide. Infect. Dis. Ther..

