# Supplementary material for: Lifetime risk of herpes zoster in the population of Beijing, China
Source: Public Health Pract (Oxf). 2023 Jan 10;5:100356. doi: 10.1016/j.puhip.2023.100356 (PMC10031117; doi:10.1016/j.puhip.2023.100356)
Supplement: Multimedia component 1 [file mmc1.docx]

# Lifetime Risk of Herpes Zoster in the Population of Beijing, China

# Supplementary Material

## Table S1. 2019 Population Estimates of Beijing Aged ≥50 Years by Age Group

| **Age (years)** | **2019 Population (10,000 persons)** |
| --- | --- |
| 50–54 | 182.8 |
| 55–59 | 150.1 |
| 60–64 | 125.3 |
| 65–69 | 84.7 |
| 70–74 | 58.7 |
| 75–79 | 42.2 |
| 80–84 | 34 |
| ≥85 | 26.4 |

## Table S2. Annual All-Cause Mortality Calculated from UNWPP 2019 Life Tables

| **Period** | **Age Group** | **Annual All-Cause Mortality^a^** |
| --- | --- | --- |
| 2015–2020 | 0 | 0.00989 |
|  | 1–4 | 0.00041 |
|  | 5–9 | 0.00031 |
|  | 10–14 | 0.00023 |
|  | 15–19 | 0.00034 |
|  | 20–24 | 0.00048 |
|  | 25–29 | 0.00064 |
|  | 30–34 | 0.00081 |
|  | 35–39 | 0.00102 |
|  | 40–44 | 0.00145 |
|  | 45–49 | 0.00213 |
|  | 50–54 | 0.00354 |
|  | 55–59 | 0.00595 |
|  | 60–64 | 0.01120 |
|  | 65–69 | 0.02024 |
|  | 70–74 | 0.03688 |
|  | 75–79 | 0.06156 |
|  | 80–84 | 0.09327 |
|  | 85–89 | 0.14233 |
|  | 90–94 | 0.19710 |
|  | 95–99 | 0.25683 |
|  | 100+ | 1 |
| 2020–2025 | 0 | 0.00839 |
|  | 1–4 | 0.00036 |
|  | 5–9 | 0.00027 |
|  | 10–14 | 0.00021 |
|  | 15–19 | 0.00031 |
|  | 20–24 | 0.00044 |
|  | 25–29 | 0.00059 |
|  | 30–34 | 0.00075 |
|  | 35–39 | 0.00095 |
|  | 40–44 | 0.00135 |
|  | 45–49 | 0.00198 |
|  | 50–54 | 0.00329 |
|  | 55–59 | 0.00551 |
|  | 60–64 | 0.01037 |
|  | 65–69 | 0.01873 |
|  | 70–74 | 0.03415 |
|  | 75–79 | 0.05727 |
|  | 80–84 | 0.08796 |
|  | 85–89 | 0.13578 |
|  | 90–94 | 0.19046 |
|  | 95–99 | 0.25093 |
|  | 100+ | 1 |
| 2025–2030 | 0 | 0.00724 |
|  | 1–4 | 0.00031 |
|  | 5–9 | 0.00024 |
|  | 10–14 | 0.00018 |
|  | 15–19 | 0.00028 |
|  | 20–24 | 0.00040 |
|  | 25–29 | 0.00054 |
|  | 30–34 | 0.00069 |
|  | 35–39 | 0.00088 |
|  | 40–44 | 0.00125 |
|  | 45–49 | 0.00184 |
|  | 50–54 | 0.00306 |
|  | 55–59 | 0.00512 |
|  | 60–64 | 0.00955 |
|  | 65–69 | 0.01725 |
|  | 70–74 | 0.03150 |
|  | 75–79 | 0.05308 |
|  | 80–84 | 0.08257 |
|  | 85–89 | 0.12956 |
|  | 90–94 | 0.18457 |
|  | 95–99 | 0.24665 |
|  | 100+ | 1 |
| 2030–2035 | 0 | 0.00632 |
|  | 1–4 | 0.00027 |
|  | 5–9 | 0.00021 |
|  | 10–14 | 0.00016 |
|  | 15–19 | 0.00025 |
|  | 20–24 | 0.00037 |
|  | 25–29 | 0.00049 |
|  | 30–34 | 0.00063 |
|  | 35–39 | 0.00081 |
|  | 40–44 | 0.00115 |
|  | 45–49 | 0.00171 |
|  | 50–54 | 0.00283 |
|  | 55–59 | 0.00474 |
|  | 60–64 | 0.00879 |
|  | 65–69 | 0.01586 |
|  | 70–74 | 0.02902 |
|  | 75–79 | 0.04928 |
|  | 80–84 | 0.07757 |
|  | 85–89 | 0.12329 |
|  | 90–94 | 0.17873 |
|  | 95–99 | 0.24240 |
|  | 100+ | 1 |
| 2035–2040 | 0 | 0.00557 |
|  | 1–4 | 0.00024 |
|  | 5–9 | 0.00018 |
|  | 10–14 | 0.00014 |
|  | 15–19 | 0.00023 |
|  | 20–24 | 0.00033 |
|  | 25–29 | 0.00045 |
|  | 30–34 | 0.00058 |
|  | 35–39 | 0.00074 |
|  | 40–44 | 0.00106 |
|  | 45–49 | 0.00159 |
|  | 50–54 | 0.00262 |
|  | 55–59 | 0.00439 |
|  | 60–64 | 0.00809 |
|  | 65–69 | 0.01456 |
|  | 70–74 | 0.02666 |
|  | 75–79 | 0.04566 |
|  | 80–84 | 0.07284 |
|  | 85–89 | 0.11725 |
|  | 90–94 | 0.17244 |
|  | 95–99 | 0.23778 |
|  | 100+ | 1 |
| 2040–2045 | 0 | 0.00495 |
|  | 1–4 | 0.00022 |
|  | 5–9 | 0.00016 |
|  | 10–14 | 0.00013 |
|  | 15–19 | 0.00020 |
|  | 20–24 | 0.00030 |
|  | 25–29 | 0.00041 |
|  | 30–34 | 0.00052 |
|  | 35–39 | 0.00068 |
|  | 40–44 | 0.00098 |
|  | 45–49 | 0.00147 |
|  | 50–54 | 0.00242 |
|  | 55–59 | 0.00405 |
|  | 60–64 | 0.00741 |
|  | 65–69 | 0.01334 |
|  | 70–74 | 0.02446 |
|  | 75–79 | 0.04217 |
|  | 80–84 | 0.06828 |
|  | 85–89 | 0.11142 |
|  | 90–94 | 0.16629 |
|  | 95–99 | 0.23258 |
|  | 100+ | 1 |
| 2045–2050 | 0 | 0.00443 |
|  | 1–4 | 0.00020 |
|  | 5–9 | 0.00014 |
|  | 10–14 | 0.00011 |
|  | 15–19 | 0.00018 |
|  | 20–24 | 0.00027 |
|  | 25–29 | 0.00037 |
|  | 30–34 | 0.00047 |
|  | 35–39 | 0.00062 |
|  | 40–44 | 0.00090 |
|  | 45–49 | 0.00136 |
|  | 50–54 | 0.00224 |
|  | 55–59 | 0.00373 |
|  | 60–64 | 0.00678 |
|  | 65–69 | 0.01220 |
|  | 70–74 | 0.02242 |
|  | 75–79 | 0.03892 |
|  | 80–84 | 0.06384 |
|  | 85–89 | 0.10572 |
|  | 90–94 | 0.16020 |
|  | 95–99 | 0.22733 |
|  | 100+ | 1 |

[a] Annual all-cause mortalities were calculated from the probabilities of dying in the UNWPP tables. UNWPP: United Nations World Population Prospects.

## Table S3. Average HZ Incidence by Age Group

| **Age (years)** | **Average HZ Incidence (per 1,000 population)** |
| --- | --- |
| 0–9 | 0.4 |
| 10–19 | 1.7 |
| 20–29 | 3.2 |
| 30–39 | 3.5 |
| 40–49 | 4.0 |
| 50–59 | 8.0 |
| 60–69 | 10.3 |
| 70–79 | 11.4 |
| ≥80 | 14.0 |

HZ: herpes zoster.

## Table S4. Average Proportion of HZ Cases Developing PHN by Age Group

| **Age (years)** | **Average Proportion of PHN Cases (%)** |
| --- | --- |
| 0–9 | 0.0% |
| 10–19 | 1.4% |
| 20–29 | 1.5% |
| 30–39 | 3.1% |
| 40–49 | 5.0% |
| 50–59 | 6.3% |
| 60–69 | 10.3% |
| 70–79 | 11.4% |
| ≥80 | 30.5% |

HZ: herpes zoster; PHN: postherpetic neuralgia.

## Table S5. Deterministic Sensitivity Analysis Parameters

| **Parameter** | **Base Case** | **Lower Value** | **Upper Value** | **Justification** |
| --- | --- | --- | --- | --- |
| Multiplier applied to current (2015–2020) annual all-cause mortality estimates (see **Table S2**) | 1 | 0.6 | 1 | Lower bound was derived through calibration; upper bound remains at 1 as Beijing LE has been well supported throughout this paper to be higher than China LE |
| Multiplier applied to future (2035–2040) annual all-cause mortality estimates (see **Table S2**) | 1 | 0.5 | 1 |  |
| Average current (2015–2020) HZ incidence^a^ in age group 0–9 years | 0.35 | 0.28 | 0.42 | ±20% |
| Average current (2015–2020) HZ incidence in age group 10–19 years | 1.65 | 1.32 | 1.98 | ±20% |
| Average current (2015–2020) HZ incidence in age group 20–29 years | 3.20 | 2.56 | 3.84 | ±20% |
| Average current (2015–2020) HZ incidence in age group 30–39 years | 3.53 | 2.82 | 4.24 | ±20% |
| Average current (2015–2020) HZ incidence in age group 40–49 years | 4.04 | 3.23 | 4.85 | ±20% |
| Average current (2015–2020) HZ incidence in age group 50–59 years | 8.03 | 6.42 | 9.64 | ±20% |
| Average current (2015–2020) HZ incidence in age group 60–69 years | 10.3 | 8.26 | 12.4 | ±20% |
| Average current (2015–2020) HZ incidence in age group 70–79 years | 11.4 | 9.13 | 13.7 | ±20% |
| Average current (2015–2020) HZ incidence in age group 80+ years | 14.0 | 11.2 | 16.8 | ±20% |
| Average future (2035–2040) HZ incidence in age group 0–9 years | 0.35 | 0.27 | 0.53 | Minimum and maximum HZ incidence was indirectly incorporated via an incidence multiplier comparing estimates derived from Jiang et al. 2019 to other East Asian locales, Taiwan and Japan^b^ |
| Average future (2035–2040) HZ incidence in age group 10–19 years | 1.65 | 1.25 | 2.48 |  |
| Average future (2035–2040) HZ incidence in age group 20–29 years | 3.20 | 3.20 | 4.80 |  |
| Average future (2035–2040) HZ incidence in age group 30–39 years | 3.53 | 3.53 | 5.30 |  |
| Average future (2035–2040) HZ incidence in age group 40–49 years | 4.04 | 4.04 | 6.06 |  |
| Average future (2035–2040) HZ incidence in age group 50–59 years | 8.03 | 8.03 | 12.1 |  |
| Average future (2035–2040) HZ incidence in age group 60–69 years | 10.3 | 10.3 | 15.5 |  |
| Average future (2035–2040) HZ incidence in age group 70–79 years | 11.4 | 11.4 | 17.1 |  |
| Average future (2035–2040) HZ incidence in age group 80+ years | 14.0 | 14.0 | 21.0 |  |
| Proportion of HZ cases with PHN in age group 0–9 years | 0 | 0 | 0.01 | Base case; base case +0.01 |
| Proportion of HZ cases with PHN in age group  10–19 years | 0.0137 | 0.01 | 0.02 | ±20% |
| Proportion of HZ cases with PHN in age group  20–29 years | 0.0145 | 0.01 | 0.02 | ±20% |
| Proportion of HZ cases with PHN in age group  30–39 years | 0.0310 | 0.02 | 0.04 | ±20% |
| Proportion of HZ cases with PHN in age group  40–49 years | 0.0504 | 0.04 | 0.06 | ±20% |
| Proportion of HZ cases with PHN in age group  50–59 years | 0.0628 | 0.05 | 0.08 | ±20% |
| Proportion of HZ cases with PHN in age group  60–69 years | 0.103 | 0.08 | 0.12 | ±20% |
| Proportion of HZ cases with PHN in age group  70–79 years | 0.114 | 0.09 | 0.14 | ±20% |
| Proportion of HZ cases with PHN in age group  80+ years | 0.305 | 0.24 | 0.37 | ±20% |

[a] Average HZ incidence was reported per 1,000 population. [b] HZ incidence estimates from Jiang et al. 2019 were compared with those from other industrialised East Asian locales as a check that they were in the same range.^1, 2^ Consequently, any differences in these incidence estimates were accounted for when calibrating the minimum and maximum HZ incidence values of the DSA. Notably, the upper bound from Japan provides an indication of future burden of disease, assuming that there is an increasing trend of HZ incidence over time. Overall, results from this comparison indicated that base case results using Jiang et al. 2019 were likely to be conservative. DSA: deterministic sensitivity analysis; HZ: herpes zoster; LE: life expectancy; PHN: postherpetic neuralgia.

## Appendix 1. Impact of Childhood Varicella Vaccination

In Beijing, a one-dose varicella vaccination (to be given at 12 months of age) was first introduced in 1997. Subsequently, a two-dose schedule vaccine (1^st^ dose at 18 months and 2^nd^ dose at 4 years) was introduced in 2002. Although varicella vaccination is still not covered under routine immunisation programs in China, a sizeable proportion of individuals still opt to receive this.^3^

This modelling exercise did not account for the potential impact of varicella vaccination on childhood varicella vaccination for the following reasons: firstly, recent large-scale analyses found no evidence that the rise of HZ incidence among older adults is linked to varicella vaccination programs.^4, 5^ The World Health Organization (WHO) also concluded that these data regarding a possible link between routine varicella vaccination in children and an increase in the HZ incidence are conflicting, and no consistent impact has been observed so far.^6, 7^

Secondly, due to the inherent nature of HZ, cases are expected to be fewer among paediatric groups and those aged <50 years, compared with those aged 50 years and above. Therefore, the potential impact of varicella vaccination on HZ cases for the relatively younger age groups was considered unlikely to significantly affect the analysis. Furthermore, this modelling exercise estimated the lifetime risk for an individual who had hypothetically lived their full life. As such, individuals aged 80 years in year 2035 would have been aged 65 in year 2020. As those in their 60s would not have received varicella vaccination, varicella vaccination is likely to have minimal to no impact on their lifetime risk of developing HZ.

Finally, although there have been some studies where the HZ incidence in vaccinated age groups decreased after varicella vaccination programs were implemented,^8^ the long-term influence of varicella vaccination on an individuals’ lifetime risk of HZ is still not well established.

Overall, given the above and the number of necessary assumptions, the present modelling exercise did not include the impact of childhood varicella vaccination.

## References

1. Chao DY, Chien YZ, Yeh YP, Hsu PS, Lian IB. The incidence of varicella and herpes zoster in Taiwan during a period of increasing varicella vaccine coverage, 2000-2008. *Epidemiol Infect* 2012;**140**(6):1131–40. 10.1017/s0950268811001786.

2. Toyama N, Shiraki K. Universal varicella vaccination increased the incidence of herpes zoster in the child-rearing generation as its short-term effect. *J Dermatol Sci* 2018;**92**(1):89–96. 10.1016/j.jdermsci.2018.07.003.

3. Zhao D, Suo L, Lu L, Pan JB, Ji WY, Liu WX. Varicella vaccine coverage before and after recommending a two-dose varicella vaccination schedule in Beijing, 2007-2017. *Chin J Vaccin Immun* 2019;**25**(2):198–202.

4. Harpaz R, Leung JW. The epidemiology of herpes zoster in the United States during the era of varicella and herpes zoster vaccines: Changing patterns among older adults. *Clin Infect Dis* 2019;**69**(2):341–4. 10.1093/cid/ciy953.

5. Wolfson LJ, Daniels VJ, Altland A, Black W, Huang W, Ou W. The Impact of varicella vaccination on the incidence of varicella and herpes zoster in the United States: Updated evidence from observational databases, 1991-2016. *Clin Infect Dis* 2020;**70**(6):995–1002. 10.1093/cid/ciz305.

6. Centers for Disease Control and Prevention: National Center for Immunization and Respiratory Diseases. Advisory Committee on Immunization Practices. *The Impact of the U.S. Varicella Vaccination Program on the Incidence of Herpes Zoster.* <https://stacks.cdc.gov/view/cdc/58913/cdc_58913_DS1.pdf?download-document-submit=Download>. [Accessed 21 December 2021].

7. Varicella and herpes zoster vaccines: WHO position paper, June 2014 – Recommendations. *Vaccine* 2014;**34**(2):198–9. 10.1016/j.vaccine.2014.07.068.

8. Weinmann S, Naleway AL, Koppolu P, Baxter R, Belongia EA, Hambidge SJ, et al. Incidence of herpes zoster among children: 2003-2014. *Pediatrics* 2019;**144**(1):e20182917. 10.1542/peds.2018-2917.
